# Maternal and umbilical cord blood polymorphonuclear leukocytes showed moderate oxidative burst at phagocytosis of *Gardnerella vaginalis*

**DOI:** 10.1186/s13104-021-05842-y

**Published:** 2021-11-22

**Authors:** Anushia Swaminathan, Nor Haslinda Abd Aziz, Najiah Ajlaa Ayub, Kon-Ken Wong, Fook-Choe Cheah

**Affiliations:** 1 Department of Paediatrics, Faculty of Medicine, Universiti Kebangsaan Malaysia, Jalan Yaacob Latif, 56000 Kuala Lumpur, Malaysia; 2 Department of Obstetrics and Gynaecology, Faculty of Medicine, Universiti Kebangsaan Malaysia, Kuala Lumpur, Malaysia; 3 Department of Pathology, Faculty of Medicine, Universiti Kebangsaan Malaysia, Kuala Lumpur, Malaysia; 4 Department of Medical Microbiology and Immunology, Faculty of Medicine, Universiti Kebangsaan Malaysia, Kuala Lumpur, Malaysia

**Keywords:** Bacterial vaginosis, Dihydrorhodamine, *E. coli*, Fluorescence, Group B Streptococcus, Hypochlorous acid, Immune response, Neutrophil, Newborn infant, Reactive oxygen species

## Abstract

**Objective:**

Pregnant women with bacterial vaginosis due to *Gardnerella vaginalis* (GV) infection presents with a wide-ranging disease symptomatology. We speculate this may be due to interaction that varies between host immune response and the pathogen. We studied the oxidative burst in polymorphonuclear leukocytes (PMNL)s from maternal blood (MB) and cord blood (CB) upon phagocytosis of GV and compared against *E. coli* and Group B Streptococcus (GBS).

**Results:**

The PHAGOBURST™ assay detects fluorescence from oxidized dihydrorhodamine during oxidative burst. The average percentage of PMNL showing oxidative burst was almost two-fold greater with GBS (99.5%) and *E. coli* (98.2%) than GV (56.9%) (p  < 0.01) in MB, but a similar proportion of PMNL with burst activity was seen in CB (84.7%). The mean fluorescence intensity (MFI) of oxidative burst in MB PMNL with GV was lower compared to *E. coli* but comparable to GBS. The MFI of CB PMNL (1580 ± 245.8) was significantly higher than MB PMNL (1198 ± 262.1) with GV, p  = 0.031. The live-cell imaging showed neutrophil oxidative burst upon phagocytosis of GV produces hypochlorous acid (HOCl). Overall, the HOCL-mediated microbicidal activity against GV is more variable and less robust than *E. coli* and GBS, especially in maternal than CB PMNL.

**Supplementary Information:**

The online version contains supplementary material available at 10.1186/s13104-021-05842-y.

## Introduction

*Gardnerella vaginalis* (GV) is a vaginal commensal commonly isolated in women of reproductive age. It is also recognised as a prevalent organism responsible for bacterial vaginosis (BV) [1]. The effects of BV are variable; from being asymptomatic to complicated pregnancies with fetal growth restriction or preterm birth [2, 3]. The host immune response to antenatal infection with GV needs further elucidation. Studies on the pathogenicity of GV are largely on epithelial cells in vitro [4–6] while information on host–pathogen interaction involving blood leukocytes is very limited. Recently, it was demonstrated that neutrophils from peripheral blood of healthy adults and amniotic fluid rapidly phagocytosed GV [7]. However, very few have studied the oxidative burst of PMNL during phagocytosis to eradicate GV. At infection sites, PMNLs are the major cells recruited and reactive oxygen species (ROS) are produced in bacterial killing. A major pathway involved is the NADPH oxidase mediated production of superoxide, hydrogen peroxide and hypochlorous acid (HOCl), the most potent antimicrobial oxidant which is myeloperoxidase (MPO) derived [8–10].

Our study sought if variability in the BV spectrum of disease among pregnant women and neonatal outcomes could be attributed to the innate host immune response during phagocytosis. We further speculate that antenatal exposure to GV may result in maternal protective immune response to the fetus, creating a difference between maternal and cord blood phagocytic responses. Our results showed a variable and modest oxidative burst especially when PMNL from maternal blood (MB) phagocytosed GV compared to *E. coli* and GBS. The live-cell imaging further showed that this is HOCl-mediated.

## Main text

### Methods

#### Subjects

Peripheral blood samples were obtained after written informed consent from 10 healthy pregnant women during antenatal clinic visits or pre-delivery at the Universiti Kebangsaan Malaysia (UKM) Medical Centre, Kuala Lumpur. The eight cord blood (CB) samples were drawn from the umbilical cord arteries after delivery of the placenta. This study was approved by the Research Ethics Committee of UKM. The clinical characteristics of the subjects are in Table 1. The neutrophil preparation and live-cell imaging were carried out at the Centre for Free Radical Research, University of Otago at Christchurch, New Zealand. A purified sample of neutrophils from the blood of a healthy laboratory staff volunteer collected for another unrelated study was used.

**Table 1:** Clinical characteristics of the mothers (n  = 10) and neonates (n  = 8)

Maternal	MB1	MB2	MB3	MB4	MB5	MB6	MB7	MB8	MB9	MB10
Age, years	38	30	40	29	35	40	42	32	37	25
Parity	2	1	3	1	3	1	3	1	2	1
Gestational age, weeks	38	40	37	38	38	38	34	38	38	39
Delivery mode	CS	CS	CS	SVD	CS	CS	CS	CS	SVD	CS
Placenta weight, g	650	460	400	600	700	650	450	750	NA	650

Neonatal	CB1	CB2	CB3	CB4	CB5	CB6	CB7	CB8
Birth weight, g	3450	3090	2640	3140	3120	3430	2870	2093
Gender	M	M	M	F	M	M	F	F
Apgar score, at 5 min	10	9	10	9	10	10	10	10

Only MB1-6 are maternal blood samples paired with the corresponding cord blood samples, CB1-6

*NA* not available; *M* male; *F* female; *CS* caesarean section; *SVD* spontaneous vaginal delivery

#### Preparation of bacteria cultures and opsonisation

The GV, ATCC^®^ 14018™ was used as the reference strain. Comparative studies utilized GBS, ATCC^®^ 13813™ and lyophilized pre-opsonised *E. coli*, provided as control in the PHAGOBURST™ kit. Columbia Blood Agar with sheep blood medium (Thermo Scientific, Melaka, Malaysia) was used for subculturing of GV and GBS, and plates were incubated in 5% CO_2_ at 37 °C. To prepare the bacterial suspensions, freshly streaked colonies were inoculated into Mueller Hinton Broth (MHB) (Thermo Scientific, Melaka, Malaysia), placed on a shaker and incubated at 37 °C overnight (24 h). The concentrations of GV and GBS suspension were adjusted to 1–2 × 10^9^/mL respectively, using the SmartSpec™ 3000 Spectrophotometer (Bio-Rad Laboratories, Hercules, CA). The opsonisation of GV and GBS utilised the serum from the corresponding MB or CB samples.

#### Oxidative burst assay

A separate aliquot of the blood sample from the MB or CB was placed in plain tubes for serum separation which were then used for opsonisation of GV and GBS following a previously published method [11]. Briefly, whole blood was centrifuged at 25 °C at 4000 rpm for 20 min. The serum was transferred into glass tubes and opsonisation of bacteria was carried out at a ratio of 1:10 of bacterial suspension to serum (vol/vol). The tubes were placed on a rotator and incubated at 37 °C for 30 min.

A 100 µL of heparinized blood was aliquoted into 5 mL tubes placed on ice (0 °C) for 10 min. Experiments were carried out according to the manufacturer’s instructions of PHAGOBURST™ (Glycotope GmbH, Heidelberg, Germany): samples of whole blood were stimulated with either 20 µL of Phorbol 12-Myristate 13-Acetate (PMA) as high stimulus, N-formyl-MetLeuPhe (fMLP) as low stimulus, pre-opsonised *E. coli* bacteria (1–2 × 10^9^/mL) as particulate/bacterial stimulus as positive controls. For comparison, the opsonised GV and GBS (each at approximately 1.5 × 10^9^/mL) prepared as above, were used as test stimuli. Samples were incubated in a water bath for 10 min at 37 °C. The samples were then assayed using BD FACSVerse™ Flow Cytometer (BD Biosciences, San Jose, CA). Data were analysed with BD FACSuite™ software (BD Biosciences, San Jose, CA) and the values were reported as the percentage of cells exhibiting fluorescent oxidised DHR of the total events and the average intensity of oxidative burst was estimated by the mean fluorescence intensity (MFI) per cell.

#### Neutrophil preparation and live-cell imaging

Neutrophils were prepared by dextran sedimentation, Ficoll-Paque (GE Healthcare, Auckland, New Zealand) centrifugation and lysis of contaminating red blood cells [12]. The neutrophil pellet is finally re-suspended in Hank’s buffered salt solution. One mM of Reagent R19-S (FutureChem Co., Seoul, Republic of Korea) stock solutions in acetonitrile were prepared fresh. R19-S reacts with HOCl to give the fluorescent product, R19 [13]. The neutrophils were placed in a 35-mm glass bottomed dish, R19-S and reagents were added 10 min prior to stimulation with opsonised GV [14]. Live-cell fluorescence emission was used to observe HOCl production. An Olympus IX-81 live-cell inverted microscope attached with an XM10 monochrome fluorescence camera and Cell R software (Olympus Soft Imaging Solutions, Munster, Germany) was used to capture time-lapse images [14].

#### Statistical analysis

Mann Whitney-U test was used to analyse the comparison between MB and CB for the percentage of cells with oxidative burst. Independent t test was used to analyse the MFI values. Comparisons between the paired MB and CB for percentage of oxidative burst used the Wilcoxon test whereas MFI values were analysed with paired t test. Data are presented as mean  ±  SEM for parametric data, median (IQR) for non-parametric data. A p value less than 0.05 was considered statistically significant. The statistical software used was GraphPad Prism Version 8.0.2 (GraphPad Software, San Diego, CA).

### Results

#### Clinical characteristics of subjects

The mean age of the women was 35 years, gestation of 38 weeks and all but two delivered by caesarean section (Table 1). All the infants had normal Apgar scores (Table 1). Their mean birth weight was 2979 g. Out of the 10 MB samples, there were only six MB-CB pairs: MB-CB1-6.

#### Oxidative burst in leukocytes

**Fig. 1:**
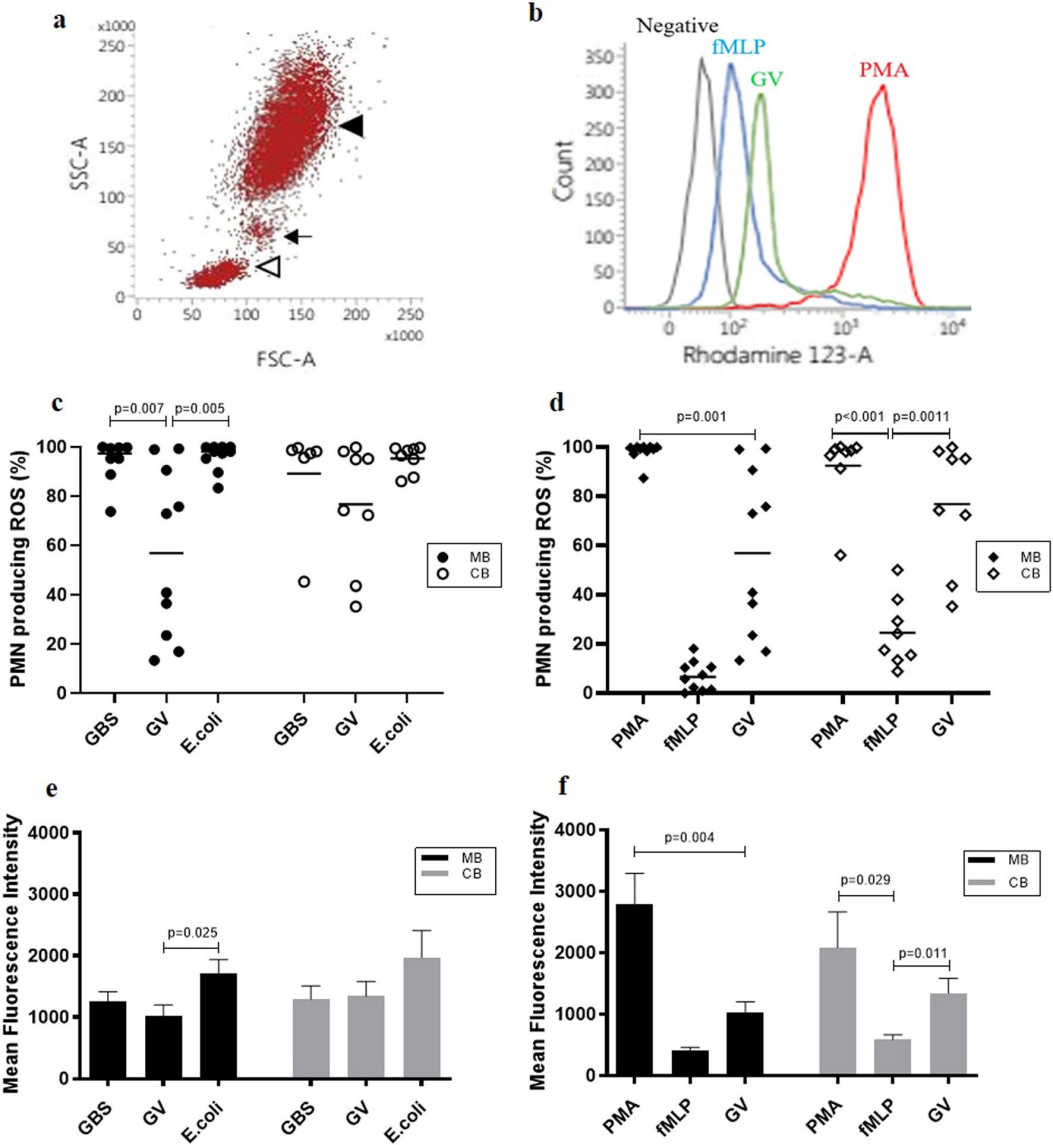
Oxidative burst responses in polymorphonuclear leukocytes (PMNLs) from maternal blood (MB) and cord blood (CB) with various stimuli. **a** Forward and side light scatter showing the leukocyte population in whole blood; black arrowhead  = PMNLs, black arrow  = Monocytes, open arrowhead  = Lymphocytes. **b** Fluorescence detection by the DHR-based oxidative burst assay on flow cytometry when PMNL are stimulated by PMA, fMLP and GV.The increased fluorescence production in PMNL when stimulated by PMA results in a significant right shift in the histogram. In comparison, fluorescence intensity is modest when the PMNL is stimulated by GV. **c** The proportion (median, IQR) of activated PMNL showing oxidative burst from MB and CB in response to the three bacteria; GBS: MB, 99.5% (95.4–99.9%), CB, 97.5% (88.9–98.8%); GV: MB, 56.9% (21.9–92.8%), CB, 84.7% (50.8–97.5%) and *E. coli*: MB, 98.2% (93.9–100.0%), CB, 97.7% (89.6–99.4%). The PMNL shows more variability in generating oxidative burst in both MB and CB with GV stimulus. This difference is more marked in the MB than CB. **d** The percentage (median, IQR) of activated PMNL with oxidative burst in MB and CB when stimulated by PMA, fMLP and GV shows the response was highest with PMA: MB, 99.5% (98.1–100%), CB, 98.4% (92.7–99.6%), followed by GV: MB, 56.9% (21.9–92.8%), CB, 84.7% (50.8–97.5%) and fMLP: MB, 6.6% (1.3–11.2%), CB, 20.9% (14.0–35.9%). **e** Intensity of oxidative burst, measured as mean fluorescence intensity (mean  ±  SEM) is highest with *E. coli*: MB (1717 ± 220.6), CB (1971 ± 441.2) than GBS: MB (1252 ± 163.4), CB (1293 ± 217.2) or GV: MB (1026 ± 176), CB (1340 ± 243.5). **f** Intensity of oxidative burst is moderate with GV: MB (1026 ± 176), CB (1340 ± 243.5) in both MB and CB, compared against the high stimulus, PMA: MB (2784 ± 509.2), CB (2081 ± 584.7) and low stimulus, fMLP: MB (400.9 ± 60.47), CB (585.5 ± 78.4). (MB, n  = 10; CB, n  = 8; with GBS, MB and CB, n  = 7 each)

The selected leukocyte population studied was PMNL based on the scatter properties in flow cytometry (Fig. 1a). Overall, oxidative burst intensity was modest in PMNL when stimulated by GV in comparison to the more robust response to PMA (Fig. 1b). Nearly all PMNL in MB showed oxidative burst when stimulated by GBS (99.5%) and *E. coli *(98.2%) as compared with GV (56.9%). There was also a wider range in the percentage of PMNL with oxidative burst at phagocytosis of GV (maximum, 99.4% to minimum, 13.4%) (Fig. 1c). The proportion of PMNL in CB showing oxidative burst when challenged with the three bacterial species, was similar with *E. coli *(97.7%) and GBS (97.5%) but lower with GV (84.7%) but the difference was not statistically significant. Even so, the PMNL in CB, as in the case with MB, showed greater variability with oxidative burst to GV, ranging between 35.3 and 99.9% (Fig. 1c). Stimulation by *E. coli *and GBS consistently showed PMNL generating oxidative bursts equivalent to PMA as a stimulus, but not with exposure to GV. However, activation by GV showed greater oxidative burst than fMLP as stimulus, in both MB and CB (Fig. 1d). With regards to the intensity of oxidative burst, MB PMNL when stimulated by GV had significantly lower MFI (1026 ± 176) than with *E. coli*, MFI (1717 ± 220.6). This was also seen in CB, although the difference was not statistically significant (Fig. 1e). Overall, the oxidative burst intensity in PMNL from MB or CB was highest with PMA and *E. coli *stimulation, moderate with GV and GBS, and was lowest with fMLP (Fig. 1f).

We further looked into the six paired MB and CB samples. The PMNL showing oxidative bursts were not different between MB and CB: *E. coli*, 99.1% vs. 97.9%; GBS, 99.5% vs 98.3% and GV, 83.2% vs 95.2%, respectively. However, the oxidative burst intensity in PMNL reacting to GV was significantly higher in CB, MFI (1580 ± 245.8) than MB, MFI (1198 ± 262.1); p  = 0.031. There was no difference in the intensity in PMNL between MB and CB when stimulated by *E. coli *or GBS.

#### Live cell-imaging of phagocytosis and oxidative burst

**Fig. 2:**
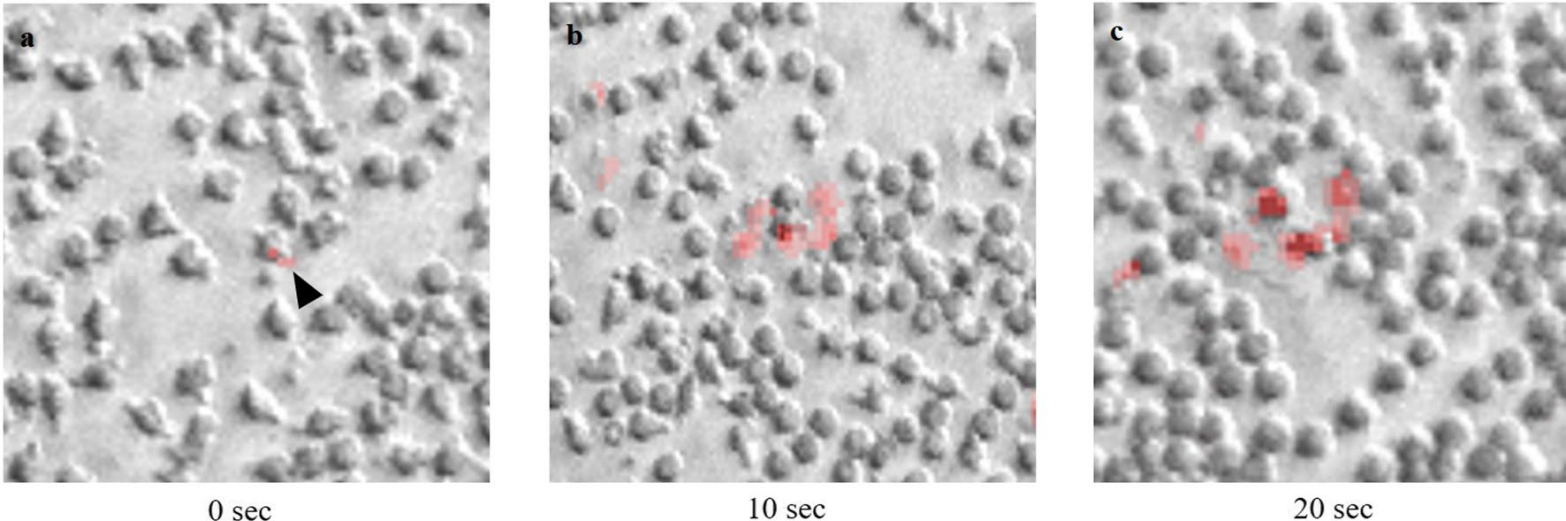
The rhodamine-based probe R19-S reacts with HOCl to yield fluorescent R19, in red (13). **a** The initial phase of oxidative burst appearing as faint red fluorescence in an activated neutrophil with pseudopod extension and GV on its surface (black arrowhead), 0 s. An increase in fluorescence intensity is observed over time and the number of neutrophils producing red fluorescence also increases but at variable intensity, **b** 10 s, and **c** 20 s. Magnification, × 20

The use of live imaging captured the phagocytosis of GV by neutrophils and fluorescence emission when HOCl is generated in the phagosome. A faint red fluorescence was seen in the beginning (0 s) (Fig. 2a), with an increasing fluorescence intensity observed over time (10 and 20 s) (Fig. 2b, c respectively). The number of neutrophils producing red fluorescence is also seen to increase but at a heterogeneous speed, followed by lowering in the intensity with quenching over time. An additional movie file shows these in more detail (see Additional file 1).

### Discussion

A seminal study by Easmon et al. reporting how peripheral blood neutrophils of healthy adults phagocytosed and killed GV was published almost 4 decades ago. [15] Although GV is the second most common organism after *Ureaplasma *sp. [16] to invade the uterine compartment to cause intrauterine infection, reports of this consequence has been limited and indicated variability of disease manifestation. For effective bacterial killing, oxidative burst during phagocytosis is a key element although some pathogens may be evasive. Unlike the robust oxidative burst by both maternal and neonatal PMNL in response to *E. coli* and GBS, the response to GV was lower and variable in quantity and quality. Whether this is the underlying reason for the variability and scarce reports of symptomatic and invasive GV disease, needs to be studied in further larger clinical trials. Of note, PMNL in the CB from our study showed a higher and less variable oxidative burst response to GV compared to MB. This is contrary to the decreased ROS production in neonatal phagocytes previously reported [17–21]. The proportion of activated PMNL, although not significantly different between MB and CB, the burst intensity appeared greater in CB. Previous studies have shown significantly higher levels of IgG in CB or neonatal blood compared to maternal circulation with up to two-fold increased IgG_1_ concentration in CB at term compared to MB [22–25]. This may explain the increased oxidative burst possibly attributed to greater opsonisation with CB samples. If this is so, it could suggest a passive form of maternal transfer of immunity to the fetus. Further studies are required to measure and compare antibody levels from mothers with GV infection and their newborn outcomes.

Our study showed that the phagocytic killing of GV is likely HOCl-mediated. Reactive oxidants are produced by the phagocyte’s NADPH oxidase enzyme, superoxide is then converted to H_2_O_2,_ which is catalysed by MPO with chloride, forms HOCl, the most powerful antimicrobial oxidant produced in the phagosome [9]. Using live cell-imaging, we could visualise the oxidative burst production by neutrophils phagocytosing GV (Additional file 1: Movie S1). Phagocytosed pathogens by neutrophils result in an increase in fluorescence, when R19-S reacts with HOCl to produce the fluorescent R19, in red [13]. Comparing the fluorescence intensity with the previous report at this lab on *Staphylococcus aureus*, [14] GV appeared to exhibit less fluorescence intensity. The lower intensity may suggest a more modest oxidative burst in the phagocytosis of GV.

In conclusion, our study demonstrated a moderately robust phagocytosis and oxidative burst effect by PMNL on GV. We also showed that PMNL oxidative burst activity was more variable in MB but more intense in CB, compared to *E. coli* and GBS. The oxidative killing of GV in PMNL is HOCl-mediated. Future studies are planned to further explore the variability in the host oxidative responses to GV of different strains and the extent of protection from circulating maternal antibodies.

## Limitations

Although uncomplicated in pregnancy, the women’s history of previous exposure to GV in particular, were not available. We did not utilise neonatal peripheral blood to obtain PMNL, especially for the live-cell imaging in view of the limited blood sample from a newborn infant that could be drawn to isolate purely neutrophils. Due to limited timing and resources, comparison of live-cell imaging results using GV was only done against the historical findings with *Staphylococcus aureus* that was recorded at the same facility [14].

## Supplementary Information

**Additional file 1: Movie S1. **The phagocytosis of GV by neutrophils and oxidative burst response captured via live-cell imaging fluorescence microscopy over a 24-sec period. Magnification, × 20.

## Data Availability

The datasets analysed in this study are available from the corresponding author upon request.

## Abbreviations

GV: *Gardnerella vaginalis*
BV: Bacterial vaginosis
ROS: Reactive oxygen species
HOCl: Hypochlorous acid
GBS: Group B *Streptococcus*
MB: Maternal blood
CB: Cord blood
PMA: Phorbol 12-Myristate 13-Acetate
fMLP: N-formyl-MetLeuPhe
DHR: Dihydrorhodamine
PMNL: Polymorphonuclear leukocyte
MPO: Myeloperoxidase
*E. coli*: *Escherichia coli*
